# Construction of Plasmid Insulin Gene Vector Containing Metallothionein IIA (pcDNAMTChIns) and Carbohydrate Response Element (ChoRE), and Its Expression in NIH3T3 Cell Line

**DOI:** 10.5812/ijem.4540

**Published:** 2012-06-30

**Authors:** Hossein Piri, Bahram Kazemi, Mohsen Rezaei, Mojgan Bandehpour, Iraj Khodadadi, Taghi Hassanzadeh, Jamshid Karimi, Fatemeh Yarian, Habibollah Peirovi, Amir Hossein Tavakoli, Mohammad Taghi Goodarzi

**Affiliations:** 1 Department of Biochemistry and Nutrition, School of Medicine, Hamadan University of Medical Science, Hamadan, IR Iran; 2 Cellular and Molecular Biology Research Center, Shahid Beheshti University of Medical Science, Tehran, IR Iran; 3 Biotechnology Department, Faculty of Medicine, Shahid Beheshti University of Medical Science, Tehran, IR Iran; 4 Nano Medicine and Tissue Engineering Research Center- Shahid Beheshti University of medical sciences, Tehran, IR Iran; 5 Iranian Tissue Bank Research and Preparation Center, Imam Khomeini Hospital Complex, Tehran University of Medical Science, Tehran, IR Iran; 6 Research Center for Molecular Medicine, Hamadan University of Medical Science, Hamadan, IR Iran

**Keywords:** Type 1 Diabetes Mellitus, Gene Therapy, Insulin

## Abstract

**Background:**

Type 1 diabetes mellitus is one of the metabolic diseases that cause insulin-producing pancreatic ß cells be destroyed by immune system self-reactive T cells. Recent­ly, new treatment methods have been developed including use of the stem cells, ß islet cells transplantation and gene therapy by viral and non-viral gene constructs.

**Objectives:**

The aim of this project was preparing the non-viral vector containing the glucose inducible insulin gene and using it in the NIH3T3 cell line.

**Materials and Methods:**

Cloning was carried out by standard methods. Total RNA was extracted from pancreatic tissue, RNA was converted to cDNA using RT-PCR reaction and preproinsulin gene was amplified using specific primers. PNMTCH plasmid was extract­ed and digested by NotI, HindIII, and MTIIA and ChoRE genes were purified and cloned into pcDNA3.1 (-) plasmid and named pcDNAMTCh. Finally, the preproinsulin genes were cloned into pcDNA3.1 (-) plasmid and pcDNAMTChIns was built.

**Results:**

The cloned gene constructs were evaluated by restriction enzyme digestion and RT-PCR. The NIH3T3 cells were transfected by plasmid naked DNA containing preproinsu­lin gene and expression was confirmed by Reverse Transcriptase PCR and Western Blot­ting Techniques.

**Conclusions:**

Gel electrophoresis of PCR products confirmed that cloning was per­formed correctly. The expression of preproinsulin gene in recombinant plasmid in NI­H3T3 cell line was observed for the first time. The findings in this study can be the basis of further research on diabetes mellitus type 1 gene therapy on animals.

## 1. Background

Type 1 diabetes mellitus (T1DM) is an autoimmune disease that immune self-reactive T cells damage the insulin producing pancreatic β-cell (1, 2) and cause insulin deficiency, hyperglycemia and long-term medical complications such as neuropathy, retinopathy and kidney failures. Exogenous insulin injection is not adequate to regulate blood glucose in normal range and causes clinical symptoms (3). Many researches carried out different surveys to achieve more effective treatments such as islet cell, stem cells or pancreas transplantation, but encountered some problems such as the risks of surgery, lack of good quality pancreas, high cost and immunological problems of the transplantation for recipients (4). Immunotherapy for autoimmune suppression is another strategy (5, 6).

Another interesting approach for T1DM treatment is viral and non-viral vectors gene therapy. The most important viral vectors include retrovirus, adenovirus and adeno associated virus. In spite of the weaknesses of the non-viral vectors, they are recently used in gene therapy researches due to their higher safety (7). However, despite all efforts in production of insulin, a proper system has not been made to provide the optimum control for glycemic patients. Since the production of insulin in human body requires exact regulation, the vectors were made for this purpose. Vectors that had the GLUT-2, glucose 6 – phosphatase and L-pyruvate kinase promoters, were tested in cell cultures and some animal tissues (8, 9). On the other hand, insulin-secreting cell lines like RIN, HIT, MIN, INS-1, βTC cells (10) and also the glucagon-like peptide-1 (GLP-1) promoter, which is in gut L-cells and is a good candidate for diabetes mellitus gene therapy, are recently used. The Special features of L-cells, suggest that L-cells and GLP-1 promoter could be useful for new approaches in diabetes gene therapy (11).

## 2. Objectives

This study was aimed to construct a non-viral vector for type 1 diabetes mellitus gene therapy. pcDNA3.1(-) eukaryotic vector containing human preproinsulin gene, metalothionein IIA (MTIIA) promoter and carbohydrates response element (ChoRE) was prepared and its responsiveness to glucose in NIH3T3 cell line was tested for the first time.

## 3. Materials and Methods

### 3.1. Total RNA Extraction From Human Pancreatic Tissue

Normal human pancreatic tissue of a brain death patient was provided from the Organ Bank of Imam Khomeini Hospital, Tehran and immediately frozen in liquid nitrogen and then kept at -80°C. Total RNA was extracted using the Total RNA extraction kit (QIAGEN) according to manufacturer’s protocol. RNA concentrations were measured using biophotometer (Ependorf) and RNA integrity was confirmed on 1.2 % agarose gel (12).

### 3.2. RT-PCR Reactions

Reverse transcription reaction (cDNA synthesis) was performed with the following materials: 1µg RNA, 1x RT buffer, 0.2 mM dNTP, 2µL random hexamer RT-Primer, 50 u MultiscribeTM RT enzyme, 4.2 µL nuclease free water in 20 µL final volume, incubated in 10 min at 25°C and 120 min at 37°C. A PCR gene amplification technique was per­formed using human preproinsulin forward and reverse primers. 

The forward primer for preproinsulin gene was 5`-AAGCTTATGGCCCTGTGGATGCGC-3` and the reverse primer was 5`-GGATCCCTAGTTGCAGTAGTTCTCCAG-3`. Primers contained HindIII and BamHI restriction sites on the 5`ends. The forward universal primer for pBluescript sk(+) plasmid was 5`-GTAAAACGACGGCCAGT-3` and the reverse primer was 5`- CAGGAAACAGCTATGAC-3`.

### 3.3. Preproinsulin Gene Cloning 

The plasmid pNMTCh containing MTIIA promoter and ChoRE was gifted by Professor O.L Kan. MTIIA promoter and ChoRE were digested by NotI and HindIII restriction enzymes, purified by gel extraction kit and ligated to NotI and HindIII digested pcDNA3.1(-) plasmid and named pcDNAMTCh.

Preproinsulin gene was amplified by PCR and ligated to pBluescript pasmid by T/A cloning method. Plasmids were transformed into Top10 E.coli strain. Bacterial colonies were cultured, plasmids were extracted and electrophoresed on 1 % agarose gel (13). 

Preproinsulin gene was digested by HindIII and Bam­HI enzymes, ligated to pcDNAMTCh and named pcD­NAMTChIns.

### 3.4. Cell Transfection

Mouse embryonic fibroblast cells (NIH3T3) were obtained from the Cell Bank of Pasteur Institute of Iran (C156). The cells were in DMEM, containing 10 % fetal calf serum (FCS), 2mM glutamine, penicillin and strep­tomycin (100 U/mL and 0.1 mg/mL respectively) at 37°C in humidified air with 5 % CO_2_ (14). NIH3T3 cells were di­vided into four groups. The first group of cells received no substance. Second group received gene free plasmid, the third group received pcDNAMTCHIns and the fourth group received plasmid pcDNAMTCHIns and 330 mM glucose. During the transfection, NIH3T3 cells covered 50-80 percent of the cultivated surface. Approximately 5×10^5^ cells per plate (5×10^5^ Cell/35 mm plate) were transfected. 3 hours before transfection, cells were incubated in 2 ml of fresh medium per 35 mm plate. Transfection solution contained 5.4 µg of plasmid DNA. This solution was incubated at room temperature for 20 minutes and then added slowly as droplets to the plates. Plates were incubated at 37 °C for 8h inCO_2_ incubator. Then the medium containing calcium - phosphate was removed and the cells were washed with culture medium. Plates were fed with 2 mL of complete growth medium and 330 mM glucose and were incubated at 37°C until analysis of gene expression completed (after 24 h). RNA was extracted using RNA purification kit and preproinsulin gene expression was examined by Reverse Transcription PCR (RT-PCR). Also, the protein product of this gene was detected by Western Blotting Technique using antibodies against human insulin and proinsulin. Firstly, proteins were separated using SDS-PAGE, then the protein bands were trans­ferred to a nitrocellulose membrane sheet. The proteins retained the same pattern of separation that they had on the gel. Uncovered surface of the membrane was blocked using milk proteins. Subsequently, the membrane was incubated with an antibody (against human proinsulin) and then, the secondary antibody conjugated with horseradish peroxidase was added. To visualize the detected bands, thesubstrate was added.

## 4. Results

**Figure 1:**
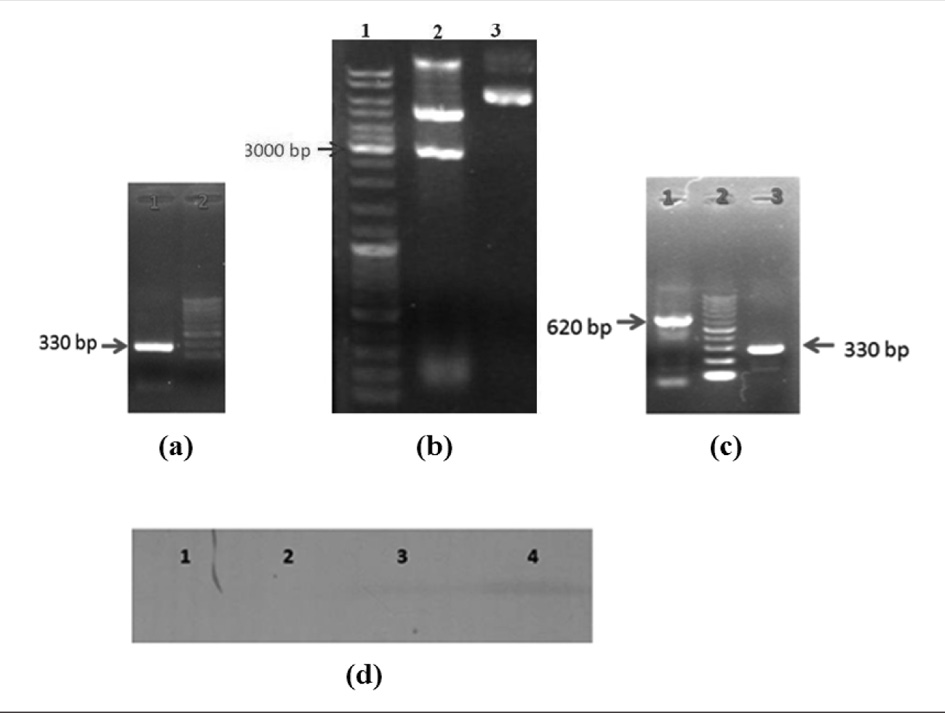
Agarose Gel Electrophoresis and Western Blotting Results a) 2 % agarose gel electrophoresis. Lane 1: PCR product of preproinsulin gene. Lane 2: DNA ladder marker (low range). b) Lane 1: DNA ladder marker. Lane 2: NotI and HindIII digested pcDNAMTCh. Lane 3: undigested pcDNAMTCh c) 2 % agarose gel electrophoresis. Lane 1: PCR product of pBluescript using plasmid universal primers. Lane 2: DNA ladder marker. Lane 3: PCR product of pBluescript using preproinsulin primers. d) Western blot analysis. Lanes 1 and 2: negative control. Lane 3: pcDNAMTChins transfected -NIH3T3 cells. Lane 4: pcDNAMTChins and 330 mM glucose – transfected NIH3T3 cells.

Total RNA was extracted from pancreatic tissue and the extracted RNA was converted to cDNA using RT- PCR reac­tion and preproinsulin gene was amplified using specific primers. PCR product (330bp) was electrophoresed on 2 % agarose gel as shown in Figure 1a. In Figure 1a, lane 1 shows the PCR product of proinsulin gene compared with the DNA ladder marker (low range). As preproinsulin gene contains 330 bp, the detected band (Figure 1a) in this re­gion confirms the PCR reaction is carried out correctly.

In Figure 1b, lane 2 is related to PNMTCH plasmid that was digested by NotI and HindIII enzymes, thus MTIIA promoter and ChoRE genes sequences were expressed together to show a band at 3000 bp region. Lane 3 shows the PNMTCH plasmid without enzyme treatment, that its length is 7817 bp. Human preproinsulin gene was cloned into pBluescript II SK (+) cloning vector and was con­firmed by PCR using specific primers for preproinsulin and plasmid universal primers. The gene free plasmid was about 280 bp in length, but the plasmid containing preproinsulin gene was about 620 bp in length that the results also proved the PCR product related to the plas­mid universal primers which was 620 bp (Figure 1c).

Preproinsulin gene was sequenced and registered to Gen­Bank at accession number JF909299.1 and is accessible in World Wide Web (http://www.ncbi.nlm.nih.gov/pubmed/) by its accession number. Preproinsulin gene was sub cloned into plasmid pcDNAMTCh and named pcDNAMTChins. The plasmid was transferred into NIH3T3 cells and its expression was confirmed using reverse transcriptase PCR (RT-PCR). The gene product was detected using qualitative western blotting technique (Figure 1d). In Figure 1d lane 1 is related to NIH3T3 cells that had received no substance, thus there is no blotting band in lane 1. Lane 2 shows the NIH3T3 cells that contained gene free plasmid, therefore no band was ob­served in blotting as expected. Lane 3 is related to the third group NIH3T3 cell line that received pcDNAMTCHIns. West­ern blotting band shows that human preproinsulin was expressed; therefore the mentioned recombinant plasmid is effective even without any glucose stimulation. The result of western blotting (lane 4) shows that human preproinsu­lin was expressed and somewhat increased by glucose (330 mM, 24h) stimulation compared to third group. These results revealed the ability of pcDNAMTCHIns to express the preproinsulin gene and to respond to glucose stimulation.

## 5. Discussion

Diabetes mellitus is one of metabolic disorders which abnormalities happen in glucose and lipid metabolism (15). Although several gene therapy studies in tissues such as exocrine pancreatic, muscle, liver, pituitary and intestinal K cells have been carried out, there is no proper system for optimal glycemic control (16). So far, many gene therapy studies have been performed on diabetes type 1, but none of them were tested on larger animals such as rabbits. In this research, non-viral vector struc­ture was prepared differently from previous vectors and tested on NIH3T3 cell line. Naked plasmid DNA structure was tested on NIH3T3 and its expression was observed for the first time. So far, different strategies have been used for diabetes treatment including functional pancreatic tissue or ß cells for transplantation in patients with type 1 diabetes and conversion of stem cells into pancreatic cells (17). According to the lack of proportionality be­tween the healthy pancreas donors and the number of patients, this method cannot be responsive to all patient requirements (4, 18). This is an important reason that has encouraged researchers to use other strategies including unlimited expression plasmids and vectors. Attempts have been made to prevent transplant rejection such as designing retroviral vector carrying IL-4 gene for ß cell Island by Kapturczak (19). His vector has an acceptable level of protein expression despite the limitations in per­formance and safety due to recombinant vector expres­sion levels (19). One of the differences of his designed vector with what we made is that its structures are viral therefore its safety is controversial in the animal body. In comparison with our non-viral construct which contains the preproinsulin and can compensate the defect in in­sulin synthesis and secretion; designed viral construct by Kapturczak targeted the immune system to improve dia­betes mellitus disease. Generally, two kinds of viral and non-viral vectors are designed and transferred to target cells. Mainly, retroviral, adeno viruses (20, 21), adeno-asso­ciated viruses (19, 22, 23) and lentiviruses (24) have been used for diabetes mellitus gene therapy. Zipris used retro­viral construction of IL-4 gene for the prevention of auto­immune diabetes (25), however there were still previous defects as mentioned about Kapturczak report. In most studies by virus vectors, there are some major problems such as unstable expression, repeated administration, stimulation of immune system and inflammation of the liver, despite positive function and high transmission efficiency (26), whereas in this project we tried to pres­ent the new efficient non-viral vector and consider the mentioned defects about vector immunogenicity. Zhang used adeno viral vector against experimental diabetes in CD-1 mice (27). Their results confirmed the viral vectors effects on inflammatory factors in the tissues such as liver. Moreover, an interesting result was also observed in their study. It was expected that insulin production in hepatocyte stimulates the glycogenesis, but the results showed otherwise, namely production of hepatic insulin inhibited the glycogen formation. It may be attributed to the cytokines. Similar results have been reported by the Hepatic Insulin Gene Therapy (HIGT) showing inhibition of glycogenesis in hepatocyte culture by high concentra­tions of glucose and insulin (21). The Dong`s report also showed the same results as that of Zhangs (27). Consid­ering the major drawbacks in the use of viral vector and using the liver as target tissue for gene therapy, we were encouraged to look for non-viral gene construct. This expression vector was constructed based on pcDNA3.1(-) eukaryotic vector containing the bifunctional promoter, metallothionein IIA (MTIIA) bound to multiple copies of carbohydrate response element (ChoRE). This study indi­cated that pcDNAMTChIns is able to induce the preproin­sulin production in the NIH3T3 cell line in the presence and absence of glucose as a stimulant. MTIIA promoter in pcDNAMTChIns plasmid has little ability to respond to glucose individually. Adding the carbohydrate response element (ChoRE) to MTIIA enhances its ability. According to presence of multiple copies of ChoRE following MTIIA, this promoter can be a proper candidate for non-viral and viral vectors and even hepatic tissues compared to GLUT-2, glucose 6 – phosphatase and L-pyruvate kinase (8, 9) and GLP-1 promoters (11). Transfer of naked plasmid DNA into NIH3T3 cell line shows this transfer is suitable and the non-viral vector can be expressed regularly by glu­cose stimulation. This finding could be the background for future success in development of the non-viral vec­tors as new gene therapy tools. Since first experimental studies have been carried out by viral and non-viral gene constructs on cell lines, the evaluation of gene constructs is a significant issue for the transition from cell lines to animals (27), thus, one of our suggestions for further re­search is studying recombinant non-viral vector on ani­mal tissues and also using the Real Time PCR for quantita­tive analysis of gene expression in cell lines and tissues.

Gene expression of preproinsulin in pcDNAMTChIns re­combinant plasmid was observed in NIH3T3 cell line for the first time that can be the basis for further research re­lated to diabetes mellitus gene therapy on animals.
